# Enhancing the methodology of clinical trials in older people: A scoping review with global perspective

**DOI:** 10.1016/j.jnha.2025.100582

**Published:** 2025-06

**Authors:** Matteo Cesari, Marco Canevelli, Wei Zhang, Jotheeswaran Amuthavalli Thiyagarajan, Domenico Azzolino, Antonio Cherubini, Jagadish K Chhetri, Amit Dias, Eduardo Ferriolli, Susanna Gentili, Celia L Gregson, Hyobum Jang, Sebastiana Kalula, Peter Lloyd-Sherlock, Radmila Matijevic, Federica Quarata, Ritu Sadana, Anshu Banerjee, Vasee Moorthy

**Affiliations:** a Ageing and Health Unit, Department of Maternal, Newborn, Child and Adolescent Health and Ageing, World Health Organization, Geneva, Switzerland; b Ageing Research Center, Department of Neurobiology, Care Sciences and Society, Karolinska Institutet and Stockholm University, Stockholm, Sweden; c National Center for Disease Prevention and Health Promotion, Italian National Institute of Health, Rome, Italy; d Department of Human Neuroscience, Sapienza University, Rome, Italy; e Research for Health Department, Science Division, World Health Organization, Geneva, Switzerland; f Geriatric Unit, Fondazione IRCCS Ca’ Granda Ospedale Maggiore Policlinico, Milan, Italy; g Geriatria, Accettazione Geriatrica e Centro di Ricerca per l'Invecchiamento, IRCCS INRCA, Ancona, Italy; h Dipartimento di Scienze Cliniche e Molecolari, Università Politecnica Delle Marche, Ancona, Italy; i National Clinical Research Center for Geriatric Diseases, Department of Geriatrics, Xuanwu Hospital, Capital Medical University, Beijing, China; j Nepalese Society of Gerontology and Geriatrics, Bhaktapur, Nepal; k Department of Preventive and Social Medicine, Goa Medical College, Goa, India; l Division of Geriatrics, Department of Internal Medicine, University of Sao Paulo Medical School, Sao Paulo, Brazil; m Global Health and Ageing Research Unit, Bristol Medical School, University of Bristol, United Kingdom; n The Health Research Unit Zimbabwe, Biomedical Research and Training Institute, Harare, Zimbabwe; o The Albertina and Walter Sisulu Institute of Ageing in Africa, Department of Medicine, University of Cape Town, Cape Town, South Africa; p Department of Nursing, Midwifery and Health, Northumbria University, Newcastle, United Kingdom; q Faculty of Medicine, University of Novi Sad, Novi Sad, Serbia; r Division of Clinical Nutrition, Department of Gastroenterology, Fondazione Policlinico Universitario Campus Bio-medico, Rome, Italy; s Department of Maternal, Newborn, Child and Adolescent Health and Ageing, World Health Organization, Geneva, Switzerland

**Keywords:** Research, Science, Randomized clinical trials, Ageing, Aging, Multimorbidity

## Abstract

As people age, they are more likely to develop chronic diseases, experience physical and mental impairments, and face social issues. This complexity makes traditional research protocols challenging, leading to the exclusion of older individuals in clinical trials (CTs) and limiting the applicability of evidence-based medicine, especially in low- and middle-income countries (LMICs).

A scoping review of the literature (based on PubMed, Embase, and Scopus) was conducted to identify recommendations to improve the methodology of CTs involving older persons. The findings were then shared with a panel of researchers with expertise in older adult research in LMICs, who assessed and refined the recommendations for implementation in low-resource settings.

After screening more than 4,700 articles, 80 were retained as relevant, providing 1,119 inputs on the design and conduct of CTs in older persons. These inputs were homogenised into 120 recommendations and organised into 13 clusters representing different phases and aspects of a CT. Key recommendations, enriched from experts’ input, indicate the importance of addressing various barriers that hinder older persons' participation in CTs in LMICs, such as poor funding, inadequate age-friendly facilities, ageism, transportation issues, and the need for standardised terminology and culturally sensitive assessment tools.

CTs involving older individuals face unique challenges. Effective methodologies and innovative approaches are essential for generating scientific evidence that informs clinical practice and promotes healthy ageing. The present work highlights the need for practical, inclusive strategies to navigate the complexities of conducting CTs in older adults.

## 1 Introduction

As people age, they are more likely to develop chronic diseases, experience physical and mental impairments, and deal with social issues. This complexity does not easily fit with the traditional standards of research protocols, first hindering the inclusion of older subjects in research studies and subsequently challenging the translation of findings into clinical practice. Additionally, it is crucial to recognise that, as people age, their needs and priorities might change, thus requiring adjustment of research goals to achieve meaningful results for older people.

In 2020, the United Nations proclaimed the Decade of Healthy Ageing (2021–2030) [1,2], a global collaboration bringing together different sectors and stakeholders to transform the world into a better place in which to grow older. The Decade is based on four action areas and four enablers [3]. One of the four enablers identifies the need to strengthen data, research, and innovation to accelerate implementation. In other words, promoting healthy ageing implies important paradigm shifts, including how we design and conduct research on ageing and involving older people.

Relatedly, a specific resolution of the 75th World Health Assembly calls on Member States to “strengthen clinical trials to provide high-quality evidence on health interventions and to improve research quality and coordination” [4]. In response to the resolution, the WHO has published the *Guidance for Best Practices for Clinical Trials* [5]. The Guidance highlights the importance of designing and implementing clinical trials (CTs) that are patient- and community-centred, leveraging innovations to enhance feasibility and effectiveness. Additionally, it advocates adopting a “risk-proportional approach” to review and monitor efficiency. The Guidance also stresses the need for more inclusive CTs, explicitly addressing the priorities of traditionally underrepresented groups in research, such as older persons.

The number of CTs has been steadily increasing, driven by the need to make clinical decisions on scientifically valid, standardised, and objective grounds. Despite this, evidence-based medicine has hitherto limited applicability to older people [6], since this age group is routinely excluded [7,8] or poorly represented [9]. Consequently, the specific health needs of older people are inadequately considered in CTs [10]. Furthermore, CTs involving older people pose unique challenges due to this population's heterogeneous and complex characteristics. It is thus critical to adopt robust methodologies and a strong rationale in CTs for practical use in promoting healthy ageing. The limited evidence from CTs involving older persons is particularly pronounced in low- and middle-income countries (LMICs), where resources, infrastructure, and staff training constrain research activities to a greater degree than in high-income countries.

This article reports the results of a scoping review aimed at identifying recommendations to improve the methodology of CTs with reference to the needs of older persons. Expecting evidence-based recommendations that largely reflect experiences from high-income countries, the article incorporates input from an international expert group specifically aimed at supporting CTs in low-resource settings, thereby allowing for a more global generalisability of research methodologies and strategies.

## 2 Methods

A scoping review was conducted to map and prioritise published recommendations on CTs methodology that are of specific relevance to older persons. The choice of applying a scoping review instead of a different review method reflected the scale and complexity of the topic. Potential domains of interest include resource requirements (e.g., staff, infrastructure, and training), inclusive eligibility criteria and processes for recruitment, defining meaningful outcomes, designing acceptable and sustainable interventions, optimising adherence and retention to the protocol, and disseminating findings. The scoping review was conducted in accordance with the Joanna Briggs Institute’s methodology for scoping reviews [11] and reported following the Preferred Reporting Items for Systematic Reviews and Meta-Analyses (PRISMA) extension for scoping reviews (PRISMA-ScR) checklist [12].

*Research question.* The scoping review aimed to address the following research question: “What are the critical recommendations for the conceptualisation, design (including how to reach older persons for recruitment), conduct, reporting, and dissemination of CTs in older persons?”.

*Search strategy and selection criteria.* A systematic search of the peer-reviewed literature was conducted using PubMed, Embase, and Scopus from inception to August 1st, 2024. These databases were prioritised as sources of multidisciplinary literature on the social and medical aspects of care for older persons. Search terms were collaboratively negotiated and approved by the research team, referring to the main area of interest, i.e., recommended methodology for CTs in older persons. The search strings used to explore the literature are reported as Supplemental Material 1. The work used specific software for literature review management (i.e., Covidence [13]) to remove duplicates and support the analysis of the entries.

Scientific articles published in English were considered of potential interest without restrictions on publication date. An article was considered of interest when it explicitly reported specific recommendations in the form of sentences or paragraphs. Guidelines, reviews, consensus articles, and position papers were the types of publications prioritised for inclusion. Recommendations provided in articles reporting the results of CTs were not considered of interest for the present work. This is motivated by their frequent inadequacy in meeting high-quality standards for research in older persons, contributing to the long-lasting evidence-based issue of geriatric medicine [[14], [15], [16]]. Their inclusion would have thus required a preliminary and comprehensive assessment of their design and capacity to conduct research in older persons.

Independent reviewers (n = 6) first screened the titles and abstracts of the articles retrieved through the literature search. At this preliminary step, a conservative approach was used, and articles that potentially included relevant information on the topic of interest were retained for more in-depth review of the full text.

In a subsequent step, pairs of reviewers independently evaluated the full texts of all potentially eligible studies. Any discrepancies were discussed and resolved by consensus. If a consensus could not be reached, a third reviewer decided whether to include the article in the final set of publications for analysis.

The following information was recorded from the included studies: (i) the journal name and where the article was published, (ii) the year of publication, and (iii) every sentence in the work referring to a potential recommendation to improve the methodology of CTs for older persons. These results were organised into clusters representing the critical phases and activities of a CT. During this process, an overarching (primary) recommendation was identified as the most commonly reported, nesting under it those (secondary) entries that were detailing aspects related to it.

*Experts from LMICs.* The scoping review's findings were shared with a gender-balanced panel of researchers (n = 7) with specific expertise in research involving older people. The experts were selected for their active coordination of studies in LMICs and their previous participation in WHO activities aimed at promoting healthy ageing. The group was asked to comment on, complete, and optimise the recommendations, considering their implementation in low-resource settings.

*Ethical aspects.* No formal approval from the Ethics Committee was necessary as the present project is based on the evaluation of existing evidence and experts’ advice.

## 3 Results

**Fig. 1:**
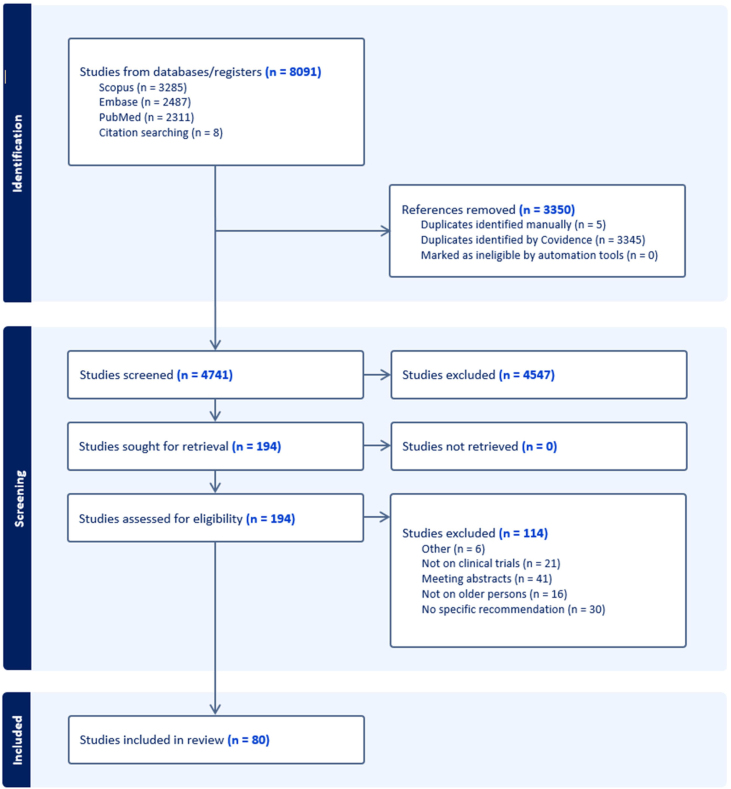
Flow chart reporting the process and results of the search strategy.

Fig. 1 presents the flow chart describing the process for retrieving articles of interest from the literature. After screening 4,741 articles and subsequent full-text analysis of 194, a final set of 80 papers was selected [7,10,[17], [18], [19], [20], [21], [22], [23], [24], [25], [26], [27], [28], [29], [30], [31], [32], [33], [34], [35], [36], [37], [38], [39], [40], [41], [42], [43], [44], [45], [46], [47], [48], [49], [50], [51], [52], [53], [54], [55], [56], [57], [58], [59], [60], [61], [62], [63], [64], [65], [66], [67], [68], [69], [70], [71], [72], [73], [74], [75], [76], [77], [78], [79], [80], [81], [82], [83], [84], [85], [86], [87], [88], [89], [90], [91], [92], [93], [94]]. Half of these (n = 40; 50.0%) provided recommendations for CT with reference to the general population of older people, while the other 40 (50.0%) were focused on selected subgroups (i.e., older persons with cancer [n = 21], sarcopenia [n = 6], cardiovascular diseases [n = 4], dementia [n = 3], COVID-19 [n = 2], depression [n = 1], infectious diseases [n = 1], osteoporosis [n = 1], and pain/palliative care [n = 1]).

The 80 articles of interest provided a total of 1,119 inputs (an average of 14.0 per article) on designing and conducting CTs in older persons. All these entries were homogenised in their wording and contents, resulting in a final set of 120 recommendations. They were subsequently organised into 13 clusters according to the phases and aspects of a CT they represented. Table 1 presents the most frequently reported recommendations organised per cluster. It is recommended, however, to consult the complete list of recommendations available in Supplemental Material 2.

**Table 1:** The most highly cited (i.e., top 20th percentile, or >17 citations) and most prevalent recommendations per aspect/phase to improve CTs involving older persons. Please refer to Supplemental Material 2 for a more comprehensive and exhaustive list of recommendations.

Aspect/Phase	Recommendations	# of citations
Organisation	Active multistakeholder engagement (including researchers, clinicians, participants, caregivers, and community representatives) in the development of the CT.*	30
	Co-design of resources and activities (including the intervention), considering cultural diversity and health literacy.*	19
	Training of research staff (including on CGA).	19
Design and Development	Consider alternative designs for the CT (for example, pragmatic trials).*	30 (19)
	Consider stratifying or enhancing the recruitment of subsets of older persons presenting specific characteristics (e.g., age, gender), levels of functional status, frailty, and/or treatment tolerance to ensure representativeness and adherence to the CT concept.	28
	Simplify and improve the accessibility of the protocol methodology.*	23
	Design of CT specifically focused on older persons (including phase II).	21
Ethical Aspects	Use short, simple consent forms and communication techniques.*	14
Eligibility Criteria	Adopt broad eligibility criteria. According to study objectives and methodology, avoid exclusion for comorbidity, disability, poor cognitive function, sensory impairment, living in residential care, and/or polypharmacy.	40
	Avoid using restrictions on eligibility based on age criterion (e.g., have no upper age limit).*	22
Recruitment	Consider using multiple channels of communication to recruit (e.g., TV ads, journals, fliers…). Involve multiple stakeholders, especially in the community. Be sure to target older persons’ relatives, general practitioners, and representatives of underserved groups.	18
Assessment	Assessment should include elements of the CGA (including morbidity).*	24 (18)
	Ensure that the functional status is adequately represented in the assessment of the participant.*	21
Intervention	The intervention should be tailored to the older person’s needs and priorities. Be sure about its feasibility.*	17
Outcome	Consider measures of physical and mental function (e.g., physical activity, physical performance measures [gait speed, SPPB], muscle strength, cognition, frailty, living independently, disability [ADL, IADL]) as an outcome of the CT.*	36
	The outcome of the CT should be meaningful for older people, also considering quality of life measures.*	27 (24)
Compliance	Support and establish good relationships with the older person and with his/her family or caregivers. Call the participant regularly; send birthday cards and “get well” cards between scheduled visits. Incentives (e.g., gadgets, prepaid gift cards, fuel vouchers, free preventive medical check-ups and examinations) can improve retention.	18
	Offer pre-planned alternatives to full clinic visits (e.g., home visits, phone calls, assessments conducted via novel technologies).	18
Adverse events	Design specific monitoring criteria for adverse events to ensure safety.	7
Data analysis	Conduct subgroup (in particular, age, gender, race, frailty) analyses.*	18
Reporting	Report the age-related characteristics of the population (e.g., age, physical and mental capacity, comorbidity, frailty, polypharmacy, living arrangement, and social support).*	10
	Report information and discuss the generalizability of the findings.*	10
Post-CT Activities	Conduct real-world evidence, registries, and post-authorisation safety and efficacy studies.	11

SPPB: Short Physical Performance Battery; ADL: Activities of Daily Living; IADL: Instrumental ADL; CT: Clinical Trial; CGA: Comprehensive Geriatric Assessment.

* Implementing the recommendation is unlikely to determine a substantial direct or indirect increase in the CT’s costs.

Table 2 includes an overview of the main comments provided by the expert group, which aimed to better consider low-resource settings and make the recommendations more feasible and acceptable in LMICs. A more detailed report of their input is included in Supplemental Material 2. Overall, experts stressed the need to consider multiple and diverse environmental barriers (e.g., poor funding, a lack of age-friendly facilities and infrastructures, ageism, adverse weather conditions, transportation issues, limited availability of ethical committees) that, more than in other national contexts, can hinder the participation of older persons in CTs. The need to standardise the nomenclature of conditions to improve clarity and consistency was also mentioned. At the same time, LMIC experts indicated the importance of adopting assessment tools that are culturally sensitive and validated for the specific setting/context where they are applied. Several inputs related to adapting recruitment strategies in low-resource settings, considering that some practices (e.g., incentives) could be ethically questionable or illegal. Another recommendation made by LMIC experts related to the importance of informing the community about proposed research activities and seeking the active engagement of critical stakeholders (in particular, older people, their families and social networks and care providers). This could increase local health literacy, ensure support for research, and improve participation in CTs.

**Table 2:** Overview of recommendations provided by the group of researchers with specific expertise on older persons in LMICs. Please refer to Supplemental Material 2 for more details.

Aspect/Phase	Recommendations
Organisation	Adequate funding is critical to support activities. Ensure the research site is age-friendly and inclusive (i.e., it considers older persons' physical and mental needs; trained staff without ageist attitudes).
Design and Development	Environmental conditions (e.g., limited accessibility to research facilities, adverse weather/seasonal conditions) can substantially affect the conduct of the CT.
	Consider methodological adaptations to reach under-represented or isolated groups (e.g., mobile teams).
	Standardise nomenclature to improve clarity and consistency.*
	Prioritise using locally validated, culturally sensitive assessment tools.*
Ethical Aspects	Strengthen the capacity and reach of ethics committees.
	Be aware of low literacy and cognitive impairment potentially affecting older people’s capacity to understand and make decisions.*
Eligibility Criteria	Avoid excluding persons based on frailty or mental capacity.
Recruitment	Undertake a period of community sensitisation before the recruitment.
	Use communication channels relevant to older persons, including those offered by local authorities.
Assessment	Assess the participant’s socioeconomic status, access to care, and health insurance status.*
	The assessment may likely identify undiagnosed or inadequately treated conditions. Plan for possible clinical follow-up and keep the GP informed. Consider alternative study designs to accommodate such possible deviations.
Intervention	Consider the participant’s expectations after the CT is over.*
Outcome	Consider the burden on family and informal caregivers.
	Do not exclusively rely on the investigator’s perceptions as potentially biased.*
Compliance	Ensure equitable access to research for persons lacking reliable transportation.
	Check local guidelines about using incentives as potentially prohibited.*
	Promote good relationships between the participants and the research staff.*
	Consider reminders of scheduled appointments.*
Adverse Events	Try to include a physician with training in geriatric care in the DSMB.*
Data Analysis	Use a representative sample for sample size calculation.
	Conduct subgroup analyses to compare rural vs urban areas.*
Reporting	Share results with the participant’s GP to ensure continuity of care.*
	Include a reflexivity statement in the scientific article. Ensure the abstract is translated into local languages. Consider writing a policy brief.*

CT: Clinical Trial; DSMB: Data and Safety Monitoring Board; GP: General Practitioner.

* Implementing the recommendation is unlikely to determine a substantial direct or indirect increase in the CT’s costs.

## 4 Discussion

The present study reports the results of a scoping review aimed at retrieving and organising the recommendations for the proper design and conduct of CTs in older persons. More than one hundred pragmatic and self-explicatory recommendations were identified. Among them, indications are provided to address several critical barriers of research involving older persons, including ageism, recruitment of high-risk participants, management of multiple health conditions and medications, how to ensure adherence to the protocol and compliance with interventions, safety, reporting adverse events, and standardised meaningful outcomes. It is also noteworthy that, to reduce the bias of a literature largely dominated by high-resource settings and deliver recommendations promoting a more global adoption of good research practice in older persons, an international panel of researchers working in LMICs commented on and completed the scoping review results.

Many of the recommendations identified in the literature are important for the successful completion of CTs involving older persons and have more general applicability. Indeed, going through the long list, several recommendations could appear as common sense. Nevertheless, each was selected and included in the document because it was perceived as frequently neglected by researchers conducting CTs. For example, a recommendation, also stressed by experts, was focused on the need to enhance the recruitment of older people into CTs, especially when testing interventions against conditions primarily affecting this age group. Although this might be logical, even recent studies conducted to test interventions against COVID-19 failed to ensure adequate representativeness of the most highly exposed population to the risk of adverse events [[94], [95], [96]]. Similarly, older persons in need of long-term care, a highly complex and vulnerable population absorbing relevant public health resources [97], are often excluded from CTs despite the lack of evidence to guide their treatments. In this context, it is worth noting that the person’s gender and frailty status were repeatedly indicated as critical variables to consider in the design and conduct of CTs, as they are too often a cause for unjustified exclusions or underrepresentation.

Due to space limitations, we selected a relatively small part of the 120 recommendations identified by our scoping review to be presented in Table 1. The selection was conducted prioritising the most commonly cited ones according to clusters. However, it should be noted that all 120 recommendations (i.e., both those listed in Table 1 and Supplemental Material 2) primarily come from consolidated literature (i.e., guidelines, task forces, consensus papers). In other words, they still have relatively strong support and methodological validity, even if only present in the Supplemental Material. Indeed, Table 1 may represent the tip of the iceberg of the broader and more detailed evidence providing many practical examples on how to address specific, but still common, issues of CTs in older persons. For this reason, we thus strongly encourage going through the more comprehensive list of recommendations to better perceive the vast opportunities to improve research on ageing and older persons. For example, whereas a single recommendation is present in Table 1 on ethical aspects (i.e., “Use short, simple consent forms and communication techniques”), Supplemental Material 2 details how to do this and additionally point out at the importance of (1) not considering inability to consent as an absolute criterion for exclusion, and (2) verifying that the older person has the ability to comply eventually conducting a formal assessment of cognition.

The biological, clinical, and social complexity frequently presented by older persons makes it critical to consider and adopt specific strategies to ensure the proper development and conduct of CTs in this population. Adequate training of research staff and time are essential for adequately assessing the older person. This becomes even more relevant when research activities are conducted in LMICs or low-resource settings where the environmental and participants’ characteristics (e.g., limited access to research facilities, low literacy, unrecognised or underdiagnosed conditions) might further challenge the methodological approach and planned activities. Evidently, this entails additional costs, which may hinder research activities involving older individuals. Furthermore, the complexity of this demographic can often result in a dilution of research findings, thereby decreasing the inclination to include them in CTs. To address these challenges, rectification is required through the implementation of specific policies and regulations that promote inclusivity and adequate representation in research. At the same time, it is worth noting that many of the recommendations identified in our work are unlikely to determine a substantial increase in direct or indirect costs of the CTs.

Among the recommendations emerging from the literature, several referred to the need to ensure age-friendly infrastructure and specifically trained personnel. Age-friendly research environments (e.g., support for transportation, facilitated access to services, proper signage, possibility of home follow-up) are critical to enabling older persons in CTs’ participation without experiencing burdens due to their possible impairments and clinical conditions. An age-friendly research environment also includes staff being adequately trained on how to consider, approach, and support older persons during all the phases of the CT. Actively addressing ageist attitudes, especially when designing the study, is critical to promote inclusiveness and enhance the quality of research. For example, many recommendations were focused on improving the definition of eligibility criteria for the CT. Deciding an individual’s eligibility based on his/her chronological age implicitly suggests an ageist approach and neglects the substantial heterogeneity of older persons. Differently, a more extensive use of objective tests to define the capacity of the person to participate and eventually benefit from the intervention is strongly recommended. In this context, a more systematic use of measures and tools traditionally part of the comprehensive geriatric assessment (i.e., a multidimensional, interdisciplinary, diagnostic, and therapeutic process designed to determine the older person’s medical, psychological, and functional needs for the development of a personalised care plan) would be helpful and should be promoted [98]. Using validated tools nested in a structured and comprehensive approach to the individual may standardise the methodology of the assessments, improve the reporting of results, and prioritise more meaningful outputs for the older person. Indeed, it is noteworthy that older persons' priorities, needs, and values may differ from those of the younger generations. For example, outcomes that are traditionally used in CTs (e.g., life expectancy, healing of the disease, improvement of a specific biological pathway) may be of limited interest for older people who may instead perceive physical and mental capacities, independent living, quality of life, or well-being to be more relevant. If the CT does not capture and incorporate the older person’s perspective, the research activity will not only generate evidence of poor translation into practice but will also impact the motivation and engagement of participants. Consistently, several recommendations point to the importance of co-designing the CT with the end-users, and initiatives today exist specifically focused on improving the definition of the relevant outcomes [99].

The adaptation of strategies for monitoring, evaluating, and reporting adverse events in CTs in older persons also emerged as a recurrent recommendation. The clinical manifestation of adverse events in older persons is frequently based on non-specific symptoms and signs, which may be challenging to be reported/documented. This can be due to (1) underreporting by the older person for a variety of reasons (e.g., poor health literacy, physical or mental impairments, self-ageism), (2) inadequate training and possible ageist attitude of the staff (e.g., potentially referring to part of “normal ageing”), and/or (3) the difficulties to disentangle new manifestations from other concurrent conditions.

Our study presents several limitations worth mentioning. Despite a comprehensive approach to retrieving the literature of interest, we prioritised recommendations from guidelines, task forces, and consensus papers published in English. Our approach assumed that these publications could effectively include the most relevant and robust evidence of interest. However, our strategy may have overlooked some related information. For example, as mentioned in the Methods section, we did not consider recommendations included in reports from CTs. Besides being counterintuitive, given that this review aims to improve quality standards for this specific type of study, including CTs’ reports would have required preliminary in-depth evaluations to ensure an adequate level of their quality to inform our recommendation list. This would have been challenging due to the risk of incomplete (and sometimes biased) reporting. Almost half of the evidence we finally analysed was focused on subgroups of older persons characterised by specific clinical conditions. For this reason, some recommendations may be more specific and relevant in specific diseases or settings. However, only 5 of the 120 recommendations were exclusively based on articles focusing on specific conditions, implicitly confirming the general validity and board applicability of the list. Finally, it is possible that a broader consultation with researchers operating in LMICs could have provided additional input to our work.

Notwithstanding these limitations, the study results provide recommendations to improve the methodology of CTs on ageing and older persons, regardless of the available resources. Rapid population ageing calls for careful reappraisal of established approaches and standards since these do not do enough to acknowledge the specific needs of older people or the complexities of including them in CTs. Developing responses that are feasible, scientifically robust and inclusive will not be an easy task. Still, it is incumbent on both public and privately funded clinical trialists to find an acceptable solution. It is critical to adopt innovative and better-calibrated methodologies for the generation of scientific evidence that can pragmatically inform clinical practice without diverting from scientific rigour.

## CRediT authorship contribution statement

MCe, MCa, WZ, and VS were in charge of the conceptualisation. MCe, MCa, WZ, and VS were in charge of the methodology. MCe, MCa, DA, AC, SG, and FQ conducted the scoping review of the literature. JC, AD, EF, CG, SK, PLS, and RM constituted the expert group that revised and improved the results of the scoping review. MCe and MCa supervised the work. MCe and MCa wrote the original draft. All authors reviewed and edited the draft, and agreed to its final version.

## Disclaimer

We alone are responsible for the views expressed in this article, and our views do not necessarily represent the views, decisions, or policies of the institutions with which we are affiliated.

## Funding

This work was funded as part of the EDCTP2 programme supported by the EU (grant number CSA2023WHO-3454-WHORCT) with funds from the UK National Institute for Health and Care Research with the use of UK aid from the UK Government to support global health research. Consultations contributed to this work was enabled by funding from the UK Department of Health and Social Care (project ID: DHSC WHO_ClinicalTrials), and Bill & Melinda Gates Foundation (grant number INV-064296).

## Declaration of competing interest

No conflict of interest was declared by the authors.
