# Supplementary material for: Enhancing the methodology of clinical trials in older people: A scoping review with global perspective
Source: J Nutr Health Aging. 2025 Jun;29(6):None. doi: 10.1016/j.jnha.2025.100582 (PMC12172962; doi:10.1016/j.jnha.2025.100582)
Supplement: Supplementary file 1 [file mmc1.pdf]

SUPPLEMENTAL MATERIAL 1

Search strings used to explore the different databases of interest.

PubMed

Filters: English language, Aged (65+ years), 80 and over: 80+ years, Humans, Date 2000-2024

(randomized controlled trial*[Title/Abstract] OR randomised controlled trial*[Title/Abstract] OR RCT[Title/Abstract] OR RCTs[Title/Abstract] OR clinical trial*[Title/Abstract])

AND

(older person*[Title/Abstract] OR older people[Title/Abstract] OR older subject*[Title/Abstract] OR older individual*[Title/Abstract] OR older participant*[Title/Abstract] OR elder*[Title/Abstract] OR older adult*[Title/Abstract] OR senior*[Title/Abstract])

AND

(guideline*[Title/Abstract] OR recommendation*[Title/Abstract] OR working group*[Title/Abstract] OR consensus[Title/Abstract] OR Delphi[Title/Abstract] OR task force*[Title/Abstract] OR workshop*[Title/Abstract] OR conference*[Title/Abstract] OR expert*[Title/Abstract] OR panel*[Title/Abstract] OR external validity[Title/Abstract])

AND

(methodology [Title/Abstract] OR design*[Title/Abstract] OR conduction[Title/Abstract] OR outcome*[Title/Abstract] OR endpoint*[Title/Abstract] OR inclusion* [Title/Abstract] OR exclusion* [Title/Abstract] OR criteri*[Title/Abstract] OR eligibility[Title/Abstract] OR intervention*[Title/Abstract] OR adherence[Title/Abstract] OR retention[Title/Abstract] OR report*[Title/Abstract])

Embase

Filters: English language, Aged (65+ years), 80 and over: 80+ years, Humans, Date 2000-2024

('randomized controlled trial*':ab,ti OR 'randomised controlled trial*':ab,ti OR 'rct':ab,ti OR 'clinical trial*':ab,ti) AND ('older person*':ab,ti OR 'older people':ab,ti OR 'older subject*':ab,ti OR 'older individual*':ab,ti OR 'older participant*':ab,ti OR 'elder*':ab,ti OR 'older adult*':ab,ti OR 'senior*':ab,ti) AND ('guideline*':ab,ti OR 'recommendation*':ab,ti OR 'working group*':ab,ti OR 'consensus':ab,ti OR 'delphi':ab,ti OR 'task force*':ab,ti OR 'workshop*':ab,ti OR 'conference*':ab,ti OR 'expert*':ab,ti OR 'panel*':ab,ti OR 'external validity':ab,ti) AND ('methodology':ab,ti OR 'design*':ab,ti OR

'conduction':ab,ti OR 'outcome*':ab,ti OR 'endpoint*':ab,ti OR 'inclusion*':ab,ti OR 'exclusion*':ab,ti OR 'criteri*':ab,ti OR 'eligibility':ab,ti OR 'intervention*':ab,ti OR 'adherence':ab,ti OR 'retention':ab,ti)

Scopus

(TITLE-ABS ("randomized controlled trial*" OR "randomised controlled trial*" OR "RCT" OR "RCTs" OR "clinical trial*") AND TITLE-ABS ("older person*" OR "older people" OR "older subject*" OR "older individual*" OR "older participant*" OR "elder*" OR "older adult*" OR "senior*") AND TITLE-ABS ("guideline*" OR "recommendation*" OR "working group*" OR "consensus" OR "Delphi" OR "task force*" OR "workshop*" OR "conference*" OR "expert*" OR "panel*" OR "external validity") AND TITLE-ABS ("methodology" OR "design*" OR "conduction" OR "outcome*" OR "endpoint*" OR "inclusion*" OR "exclusion*" OR "criteri*" OR "eligibility" OR "intervention*" OR "adherence" OR "retention" OR "report*")) AND PUBYEAR > 1999 AND PUBYEAR < 2025 AND PUBYEAR > 1999 AND PUBYEAR < 2025 AND PUBYEAR > 1999 AND PUBYEAR < 2025 AND (LIMIT-TO (LANGUAGE , "English")) AND (LIMIT-TO (EXACTKEYWORD , "Human") OR LIMIT-TO (EXACTKEYWORD , "Humans") OR LIMIT-TO (EXACTKEYWORD , "Aged"))
