# Supplementary material for: Enhancing the methodology of clinical trials in older people: A scoping review with global perspective
Source: J Nutr Health Aging. 2025 May 14;29(6):100582. doi: 10.1016/j.jnha.2025.100582 (PMC12172962; doi:10.1016/j.jnha.2025.100582)
Supplement: Supplementary file 1 [file mmc2.pdf]

SUPPLEMENTAL MATERIAL 2

Complete list of recommendations on how to conduct clinical trials in older persons. Numbered recommendations are retrieved from the scoping review of the literature and accompanied by the relevant references. Recommendations in *italics* are provided by researchers (n=7) with special expertise in clinical trials conducted in low-and-middle-income countries and involving older persons.

1. Organisation — Page 2
2. Design and Development — Page 3
3. Ethical Aspects — Page 5
4. Eligibility Criteria — Page 6
5. Recruitment — Page 7
6. Assessment — Page 8
7. Intervention — Page 10
8. Outcome — Page 11
9. Compliance — Page 12
10. Adverse Events — Page 14
11. Data Analysis — Page 15
12. Reporting — Page 16
13. Post-CT Activities — Page 17

References — Page 18

1. **Organisation**

Securing funding is crucial. Including older people in clinical trials may increase costs and complexity and potentially dilute results. This issue, particularly in low-and-middle-income countries, directly affects interest and funding clinical trials.

1.1. Consider the involvement of a multidisciplinary team (including statistical, legal and ethical experts)[1–14].
Consider incorporating research activities into the curricula of university students to enhance their training in the field and to support the conduct of clinical trials.

1.2. Active multistakeholder engagement (including researchers, clinicians, participants, caregivers, and community representatives) in the development of the clinical trial [1–9,13–33].
Consider utilising local resources and easily accessible sites to conduct the clinical trial.
   1.2.1. Co-design of resources and activities (including the intervention), considering cultural diversity and health literacy [1–3,5–7,9,13,14,18–21,23,26,27,30–32],
   1.2.2. Involvement of experts in geriatric medicine (also in the design of the intervention)[2,4,10,11,14,15,17,20,21,24,29,30,33–37],
   1.2.3. Promote diversity in the research team [9,13,19,32].

1.3. Ensure age-friendliness of the infrastructure where the clinical trial is run [3,7,9,10,13,15,18,19,21,26,29,31,36–39].
Many facilities in low-and-middle-income countries are not sufficiently equipped to meet the needs of older adults or individuals with disabilities (e.g., clearly marked toilets, facilitated access for persons with disability, etc.). This lack of accessibility may discourage participation. Age-friendly settings must be considered to address this issue.
Ensure to actively address stigma and ageism from the outset.

1.4. Training of research staff (including on the comprehensive geriatric assessment)[1–3,9,14–22,32,35–38,40]. In particular:
   1.4.1. Reduce resistance (including cultural barriers) that the staff may have to include older people in clinical trials [3,9,14,17,19,21,32,33,36,37].

2. Design and Development

2.1. Design of clinical trial specifically focused on older persons (including phase II trials)[2,4,7,14,15,20,24,26,28,30–33,37,38,41–46].
Enhancing the recruitment of older people is essential to ensure representativeness for conditions primarily affecting them.
Clinical trials should include various groups of people based on how much the condition impacts them.

2.2. Consider alternative designs for the clinical trial [4–8,10,13–16,18–20,24,25,28–34,36,37,40,41,47–50]. For example:
- 2.2.1. Pragmatic trials [6–8,10,13,15,16,20,24,28,29,33,41,47],
- 2.2.2. Adaptive trials [6,10,14,18,20,24,29,32,37],
- 2.2.3. Cluster design [5,10,27,31].

2.3. Consider conducting pilot or feasibility studies, also to test the acceptability of assessment procedures in terms of length and complexity [20,27,31,51,52].
Consider co-designing the pilot study with a relevant institution or authority to ensure methodological robustness and consistency.
When piloting and conducting the clinical trial, be mindful of the weather and seasonal conditions (e.g., snow, heat, short periods of daylight) that may affect the research activities.

2.4. Ensure that the clinical trial has an appropriate (i.e., correct balance between time for the development of clinical effect vs the risk of competing risk of death/major health event) duration of follow-up [14,25,39–41,47,49,52–55].

2.5. Incorporate real-world data (including registries) into clinical trial design [28,29,32,33,42,43,56,57].

2.6. Consider stratifying or enhancing the recruitment of subsets of older persons presenting specific characteristics (e.g., age, gender, nursing home residents), levels of functional status, frailty, and/or treatment tolerance to ensure representativeness and adherence to the clinical trialconcept [1,4,6,9,10,13,16,17,20,29,31–34,37,39,41–44,47,53,58–63].
- 2.6.1. Consider a diversity plan to ensure participation of underrepresented groups [9,13,14,28].

Certain underrepresented groups may hesitate to engage and require specific adaptations (e.g., mobile teams) to ensure participation and minimise attrition.
Consider alternative recruitment settings and data collection strategies, such as including individuals residing in long-term care facilities and conducting home assessments for those with mobility impairments.

2.7. Standardise the protocol language and training material on geriatric nomenclature [3,12,13,15,25,36,38,39,42,47,52,55,58,61,62,64,65]. In particular, be sure to define what you mean with:
- 2.7.1. Adverse events [12,52],

2.7.2. Comorbidity, multimorbidity [38],
2.7.3. Frailty [36,39,42,58,62],
2.7.4. Disability [39],
2.7.5. Sarcopenia [25,61].

Consider standardising nomenclature for other conditions and aspects critical for older persons (e.g., how diseases are diagnosed, polypharmacy, quality of life, depression, weight loss, appetite loss, physical activity) to enhance clarity and coherence. Most assessment instruments originate from high-income countries. Their appropriateness across different countries and contexts requires reassessment. For instance, standard tools may present questions or address culturally sensitive aspects. It is vital to consider locally validated versions and provide specific training to the research staff.

2.8. Simplify and improve the accessibility of the protocol methodology [1,3,5,7–9,13,14,17–21,24,36,38,41,43,48,50,59,61,66].
Consider employing diagrams, flowcharts, and checklists to enhance the research staff's comprehension of and adhesion to the protocol.

2.9. Plan to collect information about reasons for refusal to participate and withdrawals, including factors related to caregivers and major health events occurring during the follow-up [2,14,26,30,39,51,60,67].
2.10. Plan to conduct subgroup analyses (e.g., age, gender, comorbidities, frailty, physical function, cognitive function)[2,20,34,41,46,60,62,64,67,68].

3. Ethical Aspects

A significant barrier in low-and-middle-income countries is the lack of ethics committees in many areas, hindering the conduct of studies. Moreover, some existing committees may not fully adhere to national guidelines and standards. Strengthening the capacity and reach of ethics committees is crucial for ensuring oversight of ethical standards.
It is necessary to recognise specific regional differences impacting older adults in low-and-middle-income countries. Balancing ethical rigour with recommendations that might impede the feasibility of clinical trials in low-and-middle-income countries is crucial.

3.1. Use short, simple consent forms and communication techniques [3,5,7,9,13,18,19,21,22,27,38,39,48,62].

3.1.1. Employ culturally appropriate methods and use of interpreter as needed [13,19,22,32],

3.1.2. Consider sensory (i.e., hearing or vision) impairments [22,62],

3.1.3. Explain why research is relevant to older people's health and well-being [13,21],

3.1.4. Adopt multiple methods to explain the study to the participant [39,62].

Ensure and verify that the content information has been understood.
Many older adults in low-and-middle-income countries have low literacy levels and often rely on others for decision-making. This heightened dependence makes them more susceptible to misinformation or coercion into participation.

3.2. The inability to consent is not an absolute criterion for exclusion [21,33,39].
Plan for consenting methods that include those unable to consent for themselves, as they should not be routinely excluded from clinical trials.

3.3. Check understanding of critical concerns (ability to comply) and conduct a formal cognitive assessment if lacking [3,22,24,27,33,39,50,62].

3.3.1. A formal assessment of cognition should be conducted. Preferably, tests should focus on executive functioning, word fluency, and attention, not global measures of overall cognitive status [39,62].

The prevalence of dementia is rising in low-and-middle-income countries, with an estimated 90% treatment gap. Although tools for cognitive assessment are available in some local languages, the diverse range of languages and dialects in these regions presents challenges. Training personnel and expanding assessment tools to encompass more languages are essential steps. Low education levels can impact the validity of cognitive assessments. Thus, it is crucial to select tools that are culturally sensitive, educationally fair, and adapted for low-literacy environments. Ensure that the cognitive assessment is carried out using a validated tool. Verify its prior use in a comparable context and population.

3.4. Involvement of a proxy when needed, explaining his/her supporting role [9,19,21,22,24,31,38,39].

4. Eligibility Criteria

4.1. Eligibility criteria should focus on the cognitive and physical performance required to participate in the clinical trial and benefit from the intervention[5,21,39,63].

4.1.1. Elements from the comprehensive geriatric assessment might provide support [7,15,34,36,39,40,57,62].

4.2. Consider adapting eligibility criteria according to the clinical trial's objective (e.g., more selective criteria to study the mechanism of action and broader criteria to facilitate real-world representativeness)[9,20,33,40,67].

4.3. Limit exclusions to those persons who are unlikely to respond or are at risk of being harmed by the intervention. Exclusion must be fully justified [5,6,9,21,32,40,46,49,52,59,61,62,69].

4.4. Avoid using restrictions on eligibility based on age criterion (e.g., have no upper age limit)[3,6,9–11,21,22,24,32,33,36,38,39,41,43,53,57,59,62,63,70,71].

4.5. Adopt broad eligibility criteria. According to study objectives and methodology, avoid exclusion for comorbidity, disability, poor cognitive function, sensory impairment, living in residential care, and/or polypharmacy [1–3,5–10,13–15,17,19–22,24,25,29–34,36,37,39,41,43,45,47–49,59,63,71–74].
Avoid excluding individuals based on their frailty status or mental capacity (e.g., the presence of depressive symptoms).

5. **Recruitment**

5.1. Design clear recruitment strategies *a priori* [2,9,10,14,15,24,27,30].
Consider engaging in a period of community sensitisation before beginning the recruitment. In particular, increase awareness and educate people about ageism and stigma.

5.2. Consider using multiple channels of communication to recruit (e.g., TV ads, journals, fliers…). Involve multiple stakeholders, especially in the community. Be sure to target older persons' relatives, general practitioners, and representatives of underserved groups [3,6,7,9,10,13–15,19,21,24,27–29,31–33,57].
Radio remains an important communication channel for many older individuals in low-and-middle-income countries.
In most countries, clinical trials must be authorised by national regulatory agencies. Therefore, additional communication channels (e.g., government websites and flyers in public health care settings) may improve the clinical trial's visibility.

5.3. Learn from strategies adopted in previous experiences, monitor recruitment efficiency, and proactively identify barriers and facilitators (including socioeconomic factors, cultural biases, and historical mistrust)[1,11,12,15,18,19,22,28,31–33,55,75][1,8,11,12,15,18,19,22,28,31–33,55].
When relying on previous experiences, ensure they were conducted in similar socio-economic settings and apply to your current context.

5.4. Consider running multiple recruitment sites, also in non-clinical but trusted locations (e.g., places of worship, community centres), commercial venues (e.g., barbershops), or public events (e.g., cultural festivals). Ensure motivation (also through incentives for clinicians)[18,30–33,37].
Honoraria/compensation for health and care workers who support activities with their assistance may be important to consider, especially in low-resource settings.

5.5. Ensure correct communication about the clinical trial among all concerned parties (including clinicians, specialists, and older persons)[3,17,43].

6. Assessment

6.1. Consider the involvement of a multidisciplinary research team [3,4,6,11,36,37].

6.2. Consider the challenges of assessing (subjective) domains in persons who cannot self-report [56].

6.3. Consider prioritising scales and measures in the assessment, and start the evaluation with the most important ones [31].
Consider allocating sufficient time for the assessment, incorporating regular toilet breaks and refreshments breaks.

6.4. Consider the use of standardised models of comprehensive geriatric assessment [2,15,37,44,49,61,64,65,68].

6.5. Assessment should include elements of the comprehensive geriatric assessment [4,6,7,15,17,20,24,29,30,33,34,37,40,41,43–46,62,64,65,68,73,76]. In particular, ensure the following domains are adequately represented:
- 6.5.1. Functional status [1,7,15,20,24,29,30,33,34,36,37,40,46,53,62,64,65,68,69,73,76],
- 6.5.2. Cognition [15,29,30,33,34,36,37,39–41,46,53,65,76]
- 6.5.3. Nutrition [15,30,33,36,37,40,46,61,65,67–69,76],
- 6.5.4. Psychological status [15,30,34,36,37,46,65,67,76,77],
- 6.5.5. Morbidity [4,15,17,24,30,33,34,36,37,40,41,46,53,61,65,67,68,76],
- 6.5.6. Sensory deficits [41,65,76],
- 6.5.7. Social support (including evaluation of caregivers)[15,30,36,37,46,65,76],
- 6.5.8. Polypharmacy [15,33,41,46,62,65,76],
- 6.5.9. Frailty [10,24,33,42,44–46,53,58,65,67,73,76],
- 6.5.10. Self-reported health, quality of life [24,33,65],
- 6.5.11. History of falls and fractures [65,67],
- 6.5.12. Biological markers, especially renal function (i.e., creatinine clearance)[4,17,25,53,65],
- 6.5.13. Lifestyle behaviours (i.e., physical activity, diet, smoking, alcohol consumption)[40,65,67,69].

Other important areas for older people include continence, emotional well-being, and sexual health. It is also essential to assess socioeconomic status, access to healthcare (e.g., some individuals reside in rural areas and may struggle to comply), and health insurance status.

6.6. Use culturally and linguistically appropriate instruments, especially when measuring mental capacities [19,32,51].
It is essential to consider the developmental context. For instance, some older individuals in deprived areas may have never seen a watch or may not know how to read one. Therefore, some tools may not be applicable for cognitive assessment.
Many tools have not been validated in the country where they are used. When validated tools are applied in a different context or population, consider integrating a validation substudy.

6.7. Novel technologies can be considered to support the assessment. However, consider a possible digital divide and offer support if the older person is required to use them during the clinical trial [6,7,10,15,18,21,28,32,36,51].

6.8. Consider the need to act on abnormal findings potentially revealed during the clinical trial's activities (in particular, when comprehensive assessments are conducted)[4,17].
Particularly in low-and-middle-income countries, the comprehensive assessment is likely to uncover conditions that the subject was either unaware of or that were not managed appropriately. The necessity for new clinical management (for ethical reasons) may be unrelated to the clinical trial and could potentially compromise the study protocol. This may heighten the need to consider alternative designs for the clinical trial.
Keeping the general practitioner updated on all findings and outcomes resulting from the clinical trial activities is crucial.

7. Intervention

7.1. The intervention should be tailored to the older person's needs and priorities. Be sure about its feasibility [1,3,4,9,12,17,20,26,29,34,39,40,47,53,72,78].
Consider the expectations of clinical trial's participants after the study is completed.

7.2. Consider adaptations of the intervention to the heterogeneity of older persons (e.g., aligning with the general clinical recommendation "Start low and go slow")[1,4,36,40].

7.3. Consider collecting data about costs related to the intervention (e.g., implementing the intervention, delivering the comparator group intervention, health care costs, and personal out-of-pocket expenses)[55].

7.4. Document usual care (including physical exercise and healthy diet) to ensure adherence to protocol and maximise the power of comparison [31,55,67,79].
Document eventual health-related events and modifications (e.g., clinical visits and assessments, prescription changes, new diagnoses).

8. **Outcome**

8.1. The outcome of the CT should be meaningful for older people [1,2,4,8–12,15,20,21,25,29–31,33,38,40,42–44,61,63–65,72,78]. This implies the need to consider:

8.1.1. Patients reported outcomes [4,6,9,29,30,40–43,49,53,61,63,67],

8.1.2. Quality of life [1,4,8,10,20,29–31,33,38–44,55,63–65,67,68,72,80],

8.1.3. Measures of physical and mental function (e.g., physical activity, physical performance measures [gait speed, Short Physical Performance Battery], muscle strength, cognition, frailty, living independently, disability [Activities of Daily Living, Instrumental Activities of Daily Living])[1,4,6,8,10,11,20,25,29–31,33,36,38–42,44,46,49,51,53,55,61–69,72,73,76],

8.1.4. Mortality [1,20,39,49],

8.1.5. Time-related outcomes (e.g., time-to-progression, time-to-failure, time-to-benefit)[1,4,6,29,41],

8.1.6. Efficacy versus toxicity of the intervention [1,4,6],

8.1.7. Changes in social situation [4,36,42],

8.1.8. Healthcare utilisation (e.g., institutionalisation, hospitalisation)[1,8,39,49,53,63,72],

8.1.9. Burdening symptoms (e.g., depressive symptoms, pain)[6,8,33,36,77,80].

Additionally, the burden on family members and informal caregivers should be considered.

8.2. Consider elements among those included in the geriatric assessment [6,8,15,20,29,30,36,54,62,65,73].

8.3. Consider outcomes measuring the cost-effectiveness of the intervention [8,31,42,55,72].

8.4. Consider the holistic clinical impression of the investigator [39,40].
Be mindful that the impression of the investigator may be biased by many factors, especially in less rigorous institutions in developing countries.

8.5. Consider co-primary and composite outcomes when studying multidimensional geriatric conditions [29,30,54,64,66–68].

8.6. Prioritise the measurement of the primary outcome [20,39,67,73,76].

8.6.1. Consider self-reported measures as complementary outcomes [20,39,40,61,69,73].

8.6.2. Be sure to discriminate between statistically significant vs. clinically relevant results, especially when using composite scores or surrogate measures [31,40,51].

8.6.3. Prioritise outcomes unaffected by random fluctuations (e.g., choose disability lasting more than three months)[39].

8.7. Consider assessing primary outcomes through a proxy (preferably consistent throughout the study) only when the participant cannot be evaluated with other methods [39,51].

8.8. Use measures of mechanisms in the theoretical pathway between the intervention and the studied condition as secondary outcomes [39,40,66,67].

9. Compliance

9.1. Offer pre-planned alternatives to full clinic visits (e.g., home visits, phone calls, assessments conducted via novel technologies)[6,9,10,18,20,21,26,31–33,36,38,39,41,48,50–52].

9.2. Keep the burden of study visits to the minimum. Consider a flexible timeframe when scheduling evaluations [1,6–9,18–21,31,32,38,39,41,48,50,51].

9.3. Consider the possibility of integrating research activities within the usual clinical workflow [3,5,8,20,33,37].

9.4. Always consider the older person's physical and mental impairments as well as possible cultural and gender differences to adapt strategies and implement inclusive plans accordingly [3,9,10,13,14,18–21,24,26,28,31,32,36,38,48].

9.5. Consider the need to potentially provide accessible transportation [1,7,10,14,18,20–22,30,32,36,38,39,48].
A comprehensive diversity plan is essential in low-and-middle-income countries to include underrepresented groups. Inclusion and exclusion criteria must be carefully designed to ensure equitable access for older adults without reliable transportation. Mechanisms to facilitate participation, such as transport support, should be considered.
These factors should also be considered during the design phase (e.g., which to include and where the nearest assessment centre for the people in that area could be). Traffic in low-and-middle-income countries can pose a significant challenge, affecting access to research facilities and activities, especially during rush hours.

9.6. Try to reduce staff turnover. Consider assigning a staff member to guide the participant throughout the clinical trial [3,22,31,39].

9.7. Support and establish good relationships with the older person and with his/her family or caregivers. Call the participant regularly; send birthday cards and "get well" cards between scheduled visits. Incentives (e.g., gadgets, prepaid gift cards, fuel vouchers, free preventive medical check-ups and examinations) can improve retention [2,3,6,7,10,13,18,21,22,24,26,27,30–32,38,39,50].
In several countries, ethics committees may view incentives as undue inducement or coercion. Consequently, incentives may be prohibited or heavily restricted. Local regulations must be adhered to, and guidelines should be developed to ensure that incentives remain ethical and transparent.

9.8. Staff should encourage and remind of the good reasons to continue the study [3,18,32,40].
Cultivate a positive research environment. It is vital to encourage participants to adhere to the protocol.

9.9. Consider allocating sufficient time to engage with older people. Avoid interruptions during the study visit [3,5,7,9,18,21,30].

9.10. Carefully explain the research process and activities. Be sure that the participant is always kept informed [2,3,13,19,26–28,32,38].

9.11. Always prioritise the participant's safety [1,17–19,32,39].
Consider making phone calls or sending text messages to remind participants of scheduled appointments.

10. Adverse Events

10.1. Consider adverse events relevant for the older person (e.g., dizziness, falls) and atypical presentations [10,41,62,63].

10.2. Design specific monitoring criteria for adverse events to ensure safety [6,10,12,41,43,62,63].

10.3. Include geriatricians on Data and Safety Monitoring Boards [10].
Including a geriatrician in Data and Safety Monitoring Boards represents an excellent recommendation; however, the lack of geriatricians in low-and-middle-income countries may make this difficult to implement. Alternative approaches, such as training general practitioners in geriatric care, should be considered.

11. Data Analysis

It is essential to calculate the sample size carefully using a representative sample.

11.1. In the preliminary sample size calculations, consider oversampling to account for attrition and competing morbidity and mortality [5,14,20,31,33,39,40].

11.2. Develop robust statistical analysis plans, considering potential confounders and multivariable risk-based analytic methods [5,14,20,24,31,38,40,46,47,55].
Intention-to-treat analyses are advised as they tackle the common issues associated with adherence among older participants. This method is also particularly well-suited for pragmatic CTs.

11.3. Conduct subgroup (in particular, age, gender, race, frailty) analyses [6,10,14,17,22,24,33,37,41–44,46,47,57,58,64,68].
Consider additional subgroup analyses to compare urban versus rural areas.
Some ethics committees and researchers oppose the use of race in research. Consider how to appropriately evaluate this aspect.
Consider using specific comorbidities and socioeconomic status as additional criteria for stratified analyses.

12. Reporting

12.1. Adhere to the CONSORT methodology [39,74,77,81].

12.2. State the definition of the conditions of interest (e.g., frailty, disability). Report whether the effects on well-being and quality of life were ascertained [39,58].

12.3. Report the age-related characteristics of the population (e.g., age, physical and mental capacity, comorbidity, frailty, polypharmacy, living arrangement, and social support)[10,11,22,24,31,37,47,68,71,81].

12.4. Report the sample size calculations and how the drop-out expected rates were factored [39].

12.5. Discuss ethical issues and procedures when reporting results [39].

12.6. Describe strategies to reduce exclusions and attrition and provide details for the eventual missing data (including their handling)[31,39,47,55].

12.7. Report information and discuss the generalizability of the findings [2,10,20,24,31,39,47,47,71,74].

12.8. Consider reporting information about the value for money of the intervention [55].

12.9. Communication of age- and frailty-specific knowledge and evidence gaps [1,10,25,58,68].

12.10. Share study results with community stakeholders and older persons [13,27,32].
Make sure to share the results also with caregivers.
Involve general practitioners in reporting clinical trial outcomes of their patients to ensure continuity of care and enhance decision-making.
When publishing a scientific paper, include a reflexivity statement and ensure that the abstract is accessible in the local languages of all participants.
Consider drafting the results as a policy brief, particularly for dissemination to policymakers.

13. Post-Clinical Trial Activities

13.1. Conduct real-world evidence, registries, and post-authorisation safety and efficacy studies [10,29,30,33,41,42,44,47,53,64,68].

Note. The following studies were specifically focused on:

- Cancer: [1-4,7,8,15-17,20,29,30,34,36,44,46,53,64,68,74,78],
- COVID-19: [9,10],
- Cardiovascular diseases: [45,58,60,72],
- Dementia: [51,52,71],
- Depression: [77],
- Infectious disease: [11],
- Osteoporosis: [49],
- Pain and palliative care: [80],
- Sarcopenia: [25,40,61,66,67,69].

REFERENCES

1 Kilari D, Soto-Perez-de-Celis E, Mohile SG, *et al.* Designing exercise clinical trials for older adults with cancer: Recommendations from 2015 Cancer and Aging Research Group NCI U13 Meeting. *J Geriatr Oncol*. 2016;7:293–304. doi: 10.1016/j.jgo.2016.04.007

2 Freedman RA, Dockter TJ, Lafky JM, *et al.* Promoting Accrual of Older Patients with Cancer to Clinical Trials: An Alliance for Clinical Trials in Oncology Member Survey (A171602). *Oncologist*. 2018;23:1016–23. doi: 10.1634/theoncologist.2018-0033

3 BrintzenhofeSzoc K, Canin B, Casas-Silva E, *et al.* Through the Lens of Patient Partners: Challenges in Accrual of Older Adults to NCI Clinical Trials. *J Natl Cancer Inst Monogr*. 2022;2022:125–34. doi: 10.1093/jncimonographs/lgac022

4 Pallis AG, Fortpied C, Wedding U, *et al.* EORTC elderly task force position paper: Approach to the older cancer patient. *Eur J Cancer*. 2010;46:1502–13. doi: 10.1016/j.ejca.2010.02.022

5 Choong EKM, Gallagher JE, Patel R. Methodology and outcome of trials involving older adults in UK care homes: A rapid review. *Gerodontology*. 2023;40:1–9. doi: 10.1111/ger.12622

6 Habr D, McRoy L, Papadimitrakopoulou VA. Age Is Just a Number: Considerations for Older Adults in Cancer Clinical Trials. *J Natl Cancer Inst*. 2021;113:1460–4. doi: 10.1093/jnci/djab070

7 Hopkins JO, Braun-Inglis C, Guidice S, *et al.* Enrolling Older Adults Onto National Cancer Institute-Funded Clinical Trials in Community Oncology Clinics: Barriers and Solutions. *J Natl Cancer Inst Monogr*. 2022;2022:117–24. doi: 10.1093/jncimonographs/lgac019

8 Nipp RD, Yao N (Aaron), Lowenstein LM, *et al.* Pragmatic study designs for older adults with cancer: Report from the U13 conference. *J Geriatr Oncol*. 2016;7:234–41. doi: 10.1016/j.jgo.2016.02.005

9 Witham MD, Anderson E, Carroll CB, *et al.* Ensuring that COVID-19 research is inclusive: guidance from the NIHR INCLUDE project. *BMJ Open*. 2020;10:e043634. doi: 10.1136/bmjopen-2020-043634

10 Andrew MK, Schmader KE, Rockwood K, *et al.* Considering Frailty in SARS-CoV-2 Vaccine Development: How Geriatricians Can Assist. *Clin Interv Aging*. 2021;16:731–8. doi: 10.2147/CIA.S295522

11 High KP, Bradley S, Loeb M, *et al.* A New Paradigm for Clinical Investigation of Infectious Syndromes in Older Adults: Assessment of Functional Status as a Risk Factor and Outcome Measure. *Clin Infect Dis*. 2005;40:114–22. doi: 10.1086/426082

12 Baim-Lance A, Ferreira KB, Cohen HJ, *et al.* Improving the Approach to Defining, Classifying, Reporting and Monitoring Adverse Events in Seriously Ill Older Adults: Recommendations from a Multi-stakeholder Convening. *J Gen Intern Med*. 2023;38:399–405. doi: 10.1007/s11606-022-07646-7

13 Samper-Ternent R, Silveira SL, Stevens A, *et al.* Considerations When Designing and Implementing Pragmatic Clinical Trials That Include Older Hispanics. *Ethn Dis*. 2024;33:76–83. doi: 10.18865/ed.33.2-3.076

14 Petrovsky DV, Đoàn LN, Loizos M, *et al.* Key recommendations from the 2021 “inclusion of older adults in clinical research” workshop. *J Clin Transl Sci*. 2022;6:e55. doi: 10.1017/cts.2022.1

15 Kimmick G, Sedrak MS, Williams G, *et al.* Infrastructure to Support Accrual of Older Adults to National Cancer Institute Clinical Trials. *JNCI Monographs*. 2022;2022:151–8. doi: 10.1093/jncimonographs/lgac025

16 Sridhara R, Marchenko O, Jiang Q, *et al.* Evaluation of Treatment Effect in Underrepresented Population in Cancer Trials: Discussion with International Regulators. *Stat Biopharm Res*. 2023;15:450–6. doi: 10.1080/19466315.2022.2128404

17 Aapro MS, Köhne C-H, Cohen HJ, *et al.* Never Too Old? Age Should Not Be a Barrier to Enrollment in Cancer Clinical Trials. *Oncologist*. 2005;10:198–204. doi: 10.1634/theoncologist.10-3-198

18 Buttgereit T, Palmowski A, Forsat N, *et al.* Barriers and potential solutions in the recruitment and retention of older patients in clinical trials—lessons learned from six large multicentre randomized controlled trials. *Age Ageing*. 2021;50:1988–96. doi: 10.1093/ageing/afab147

19 Chopde S. Promoting Inclusion in Clinical Trials: A Rapid Review of the Literature and Recommendations for Action. *Int J Pharm Clin Res*. 2023;15:487–93.

20 Le-Rademacher J, Mohile S, Unger J, *et al.* Trial Design Considerations to Increase Older Adult Accrual to National Cancer Institute Clinical Trials. *J Natl Cancer Inst Monogr*. 2022;2022:135–41. doi: 10.1093/jncimonographs/lgac023

21 Goodwin VA, Low MSA, Quinn TJ, *et al.* Including older people in health and social care research: best practice recommendations based on the INCLUDE framework. *Age Ageing*. 2023;52:afad082. doi: 10.1093/ageing/afad082

22 Herrera AP, Snipes SA, King DW, *et al.* Disparate Inclusion of Older Adults in Clinical Trials: Priorities and Opportunities for Policy and Practice Change. *Am J Public Health*. 2010;100:S105–12. doi: 10.2105/AJPH.2009.162982

23 Travers J, Romero-Ortuno R, Ní Shé É, *et al.* Involving older people in co-designing an intervention to reverse frailty and build resilience. *Family Practice*. 2022;39:200–6. doi: 10.1093/fampra/cmab084

24 Vaughan CP, Dale W, Allore HG, *et al.* AGS Report on Engagement Related to the NIH Inclusion Across the Lifespan Policy. *J Am Geriatr Soc*. 2019;67:211–7. doi: 10.1111/jgs.15784

25 Cesari M, Fielding R, Pahor M, *et al.* Biomarkers of sarcopenia in clinical trials-recommendations from the International Working Group on Sarcopenia. *J Cachexia Sarcopenia Muscle*. 2012;3:181–90. doi: 10.1007/s13539-012-0078-2

26 Chao D, Foy CG, Farmer D. Exercise Adherence among Older Adults: Challenges and Strategies. *Control Clin Trials*. 2000;21:S212–7. doi: 10.1016/S0197-2456(00)00081-7

27 Huang L, Lü J, Chen N, *et al.* Recruitment of older adults into randomized controlled trials: Issues and lessons learned from two community-based exercise interventions in Shanghai. *J Sport Health Sci*. 2016;5:308–14. doi: 10.1016/j.jshs.2016.07.009

28 Masters JC, Cook JA, Anderson G, *et al.* Ensuring diversity in clinical trials: The role of clinical pharmacology. *Contemp Clin Trials*. 2022;118:106807. doi: 10.1016/j.cct.2022.106807

29 Sedrak MS, Freedman RA, Cohen HJ, *et al.* Older adult participation in cancer clinical trials: A systematic review of barriers and interventions. *CA Cancer J Clin*. 2021;71:78–92. doi: 10.3322/caac.21638

30 Singh H, Hurria A, Klepin HD. Progress Through Collaboration: An ASCO and U.S. Food and Drug Administration Workshop to Improve the Evidence Base for Treating Older Adults With Cancer. *Am Soc Clin Oncol Educ Book*. 2018;392–9. doi: 10.1200/EDBK_201133

31 Pitkala KH, Strandberg TE. Clinical trials in older people. *Age Ageing*. 2022;51:afab282. doi: 10.1093/ageing/afab282

32 Enhancing the Diversity of Clinical Trial Populations Eligibility Criteria, Enrollment Practices, and Trial Designs. Guidance for Industry. US Department of Health and Human Services 2020.

33 Liu Q, Schwartz JB, Slattum PW, *et al.* Roadmap to 2030 for Drug Evaluation in Older Adults. *Clin Pharmacol Ther*. 2022;112:210–23. doi: 10.1002/cpt.2452

34 Satariano WA, Silliman RA. Comorbidity: implications for research and practice in geriatric oncology. *Crit Rev Oncol Hematol*. 2003;48:239–48. doi: 10.1016/j.critrevonc.2003.08.002

35 Newman JC, Sokoloski JL, Robbins PD, *et al.* Creating the Next Generation of Translational Geroscientists. *J Am Geriatr Soc*. 2019;67:1934–9. doi: 10.1111/jgs.16055

36 Hurria A, Dale W, Mooney M, *et al.* Designing Therapeutic Clinical Trials for Older and Frail Adults With Cancer: U13 Conference Recommendations. *J Clin Oncol*. 2014;32:2587–94. doi: 10.1200/JCO.2013.55.0418

37 Hurria A, Levit LA, Dale W, *et al.* Improving the Evidence Base for Treating Older Adults With Cancer: American Society of Clinical Oncology Statement. *J Clin Oncol*. 2015;33:3826–33. doi: 10.1200/JCO.2015.63.0319

38 Chung J-Y. Geriatric clinical pharmacology and clinical trials in the elderly. *Transl Clin Pharmacol*. 2014;22:64–9. doi: 10.12793/tcp.2014.22.2.64

39 Ferrucci L, Guralnik J, Studenski S, *et al.* Designing randomized, controlled trials aimed at preventing or delaying functional decline and disability in frail, older persons: a consensus report. *J Am Geriatr Soc*. 2004;52:625–34. doi: 10.1111/j.1532-5415.2004.52174.x

40 Bhasin S, Cawthon PM, Correa-de-Araujo R, *et al.* Optimizing the Design of Clinical Trials to Evaluate the Efficacy of Function-Promoting Therapies. *J Gerontol A Biol Sci Med Sci*. 2023;78:86–93. doi: 10.1093/gerona/glad024

41 Walker LE, Pirmohamed M. The status of drug evaluation in older adults in the United Kingdom: Bridging the representation gap. *J Am Geriatr Soc*. 2024;n/a. doi: 10.1111/jgs.18817

42 Liau SJ, Lalic S, Sluggett JK, *et al.* Medication Management in Frail Older People: Consensus Principles for Clinical Practice, Research, and Education. *J Am Med Dir Assoc*. 2021;22:43–9. doi: 10.1016/j.jamda.2020.05.004

43 Bumanlag IM, Jaoude JA, Rooney MK, *et al.* Exclusion of Older Adults from Cancer Clinical Trials: Review of the Literature and Future Recommendations. *Semin Radiat Oncol*. 2022;32:125–34. doi: 10.1016/j.semradonc.2021.11.003

44 Kaźmierska J. Do we protect or discriminate? Representation of senior adults in clinical trials. *Rep Pract Oncol Radiother*. 2013;18:6–10. doi: 10.1016/j.rpor.2012.08.006

45 Wierzbicki AS. Preventive cardiology for the aging population: how can we better design clinical trials of statins? *Expert Rev Cardiovasc Ther*. 2024;22:13–8. doi: 10.1080/14779072.2024.2302122

46 Hurria A, Cohen H, Extermann M. Geriatric oncology research in the cooperative groups: A report of a SIOG special meeting. *J Geriatr Oncol*. 2010;1:40–4. doi: 10.1016/j.jgo.2010.03.005

47 Kennedy-Martin T, Curtis S, Faries D, *et al.* A literature review on the representativeness of randomized controlled trial samples and implications for the external validity of trial results. *Trials*. 2015;16. doi: 10.1186/s13063-015-1023-4

48 Cherubini A, Oristrell J, Pla X, *et al.* The persistent exclusion of older patients from ongoing clinical trials regarding heart failure. *Arch Intern Med*. 2011;171:550–6. doi: 10.1001/archinternmed.2011.31

49 Rolland Y, Cesari M, Fielding RA, *et al.* Osteoporosis in Frail Older Adults: Recommendations for Research from the ICFSR Task Force 2020. *J Frailty Aging*. 2021;10:168–75. doi: 10.14283/jfa.2021.4

50 Lacey RJ, Wilkie R, Wynne-Jones G, *et al.* Evidence for strategies that improve recruitment and retention of adults aged 65 years and over in randomised trials and observational studies: a systematic review. *Age Ageing*. 2017;46:895–903. doi: 10.1093/ageing/afx057

51 Andrieu S, Coley N, Aisen P, *et al.* Methodological issues in primary prevention trials for neurodegenerative dementia. *J Alzheimers Dis*. 2009;16:235–70. doi: 10.3233/JAD-2009-0971

52 Salzman C, Jeste DV, Meyer RE, *et al.* Elderly patients with dementia-related symptoms of severe agitation and aggression: consensus statement on treatment options, clinical trials methodology, and policy. *J Clin Psychiatry*. 2008;69:889–98. doi: 10.4088/jcp.v69n0602

53 Denkinger M, Knol W, Cherubini A, *et al.* Inclusion of functional measures and frailty in the development and evaluation of medicines for older adults. *Lancet Healthy Longev*. 2023;4:e724–9. doi: 10.1016/S2666-7568(23)00208-8

54 de Souto Barreto P, Cesari M, Morley JE, *et al.* Appetite Loss and Anorexia of Aging in Clinical Care: An ICFSR Task Force Report. *J Frailty Aging*. 2022;11:129–34. doi: 10.14283/jfa.2022.14

55 Davis JC, Robertson MC, Comans T, *et al.* Guidelines for conducting and reporting economic evaluation of fall prevention strategies. *Osteoporos Int*. 2011;22:2449–59. doi: 10.1007/s00198-010-1482-0

56 Ersek M, Polissar N, Pen AD, *et al.* Addressing methodological challenges in implementing the nursing home pain management algorithm randomized controlled trial. *Clin Trials*. 2012;9:634–44. doi: 10.1177/1740774512454243

57 Florisson S, Aagesen EK, Bertelsen AS, *et al.* Are older adults insufficiently included in clinical trials?—An umbrella review. *Basic Clin Pharmacol Toxicol*. 2021;128:213–23. doi: 10.1111/bcpt.13536

58 Cerreta F, Padrão A, Skibicka-Stepien I, *et al.* Medicines for older people: assessment and transparency at the European Medicines Agency regarding cardiovascular and antithrombotic medicinal products. *Eur Geriatr Med*. 2018;9:415–8. doi: 10.1007/s41999-018-0071-1

59 Markham SC, McNab J, O'Loughlin K, *et al.* International responses addressing the under-representation of older people in clinical research. *Australas J Ageing*. 2023;42:762–8. doi: 10.1111/ajag.13234

60 Schwartz JB, Schmader KE, Hanlon JT, *et al.* Pharmacotherapy in Older Adults with Cardiovascular Disease: Report from an American College of Cardiology, American Geriatrics Society, and National Institute on Aging Workshop. *J Am Geriatr Soc*. 2019;67:371–80. doi: 10.1111/jgs.15634

61 Vellas B, Pahor M, Manini T, *et al.* Designing pharmaceutical trials for sarcopenia in frail older adults: EU/US task force recommendations. *J Nutr Health Aging*. 2013;17:612–8. doi: 10.1007/s12603-013-0362-7

62 Diener L, Hugonot-Diener L, Alvino S, *et al.* Guidance synthesis. Medical research for and with older people in Europe: Proposed ethical guidance for good clinical practice: Ethical considerations. *J Nutr Health Aging*. 2013;17:625–7. doi: 10.1007/s12603-013-0340-0

63 Vitale C, Fini M, Spoletini I, *et al.* Under-representation of elderly and women in clinical trials. *Int J Cardiol*. 2017;232:216–21. doi: 10.1016/j.ijcard.2017.01.018

64 Pallis AG, Ring A, Fortpied C, *et al.* EORTC workshop on clinical trial methodology in older individuals with a diagnosis of solid tumors. *Ann Oncol*. 2011;22:1922–6. doi: 10.1093/annonc/mdq687

65 Van Kan GA, Sinclair A, Andrieu S, *et al.* The geriatric minimum data set for clinical trials (GMDS). *J Nutr Health Aging*. 2008;12:197–200. doi: 10.1007/BF02982620

66 Cooper C, Fielding R, Visser M, *et al.* Tools in the assessment of sarcopenia. *Calcif Tissue Int*. 2013;93:201–10. doi: 10.1007/s00223-013-9757-z

67 Reginster J-Y, Beaudart C, Al-Daghri N, *et al.* Update on the ESCEO recommendation for the conduct of clinical trials for drugs aiming at the treatment of sarcopenia in older adults. *Aging Clin Exp Res*. 2021;33:3–17. doi: 10.1007/s40520-020-01663-4

68 Wildiers H, Brain E, Penninckx B, *et al.* The EORTC Cancer in the Elderly Task Force, a Protostar for EORTC's future. *EurJ Cancer Suppl*. 2012;10:34–8. doi: 10.1016/S1359-6349(12)70008-3

69 Correa-de-Araujo R, Bhasin S. Public Health Need, Molecular Targets, and Opportunities for the Accelerated Development of Function-Promoting Therapies: Proceedings of a National Institute on Aging Workshop. *J Gerontol A BIol Sci Med Sci*. 2022;77:2227–37. doi: 10.1093/gerona/glac181

70 Hughes CM, Cadogan CA, Patton D, *et al.* Pharmaceutical strategies towards optimising polypharmacy in older people. *Int J Pharm*. 2016;512:360–5. doi: 10.1016/j.ijpharm.2016.02.035

71 Leinonen A, Koponen M, Hartikainen S. Systematic Review: Representativeness of Participants in RCTs of Acetylcholinesterase Inhibitors. *PLoS One*. 2015;10:e0124500. doi: 10.1371/journal.pone.0124500

72 Rich MW, Chyun DA, Skolnick AH, *et al.* Knowledge Gaps in Cardiovascular Care of the Older Adult Population: A Scientific Statement From the American Heart Association, American College of Cardiology, and American Geriatrics Society. *J Am Coll Cardiol*. 2016;67:2419–40. doi: 10.1016/j.jacc.2016.03.004

73 Functional Outcomes for Clinical Trials in Frail Older Persons: Time To Be Moving: Working Group on Functional Outcome Measures for Clinical Trials*. *J Gerontol A Biol Sci Med Sci*. 2008;63:160–4. doi: 10.1093/gerona/63.2.160

74 Battisti NML, Sehovic M, Extermann M. Assessment of the External Validity of the National Comprehensive Cancer Network and European Society for Medical Oncology Guidelines for Non-Small-Cell Lung Cancer in a Population of Patients Aged 80 Years and Older. *Clin Lung Cancer*. 2017;18:460–71. doi: 10.1016/j.cllc.2017.03.005

75 Nipp RD, Yao N (Aaron), Lowenstein LM, *et al.* Pragmatic study designs for older adults with cancer: Report from the U13 conference. *J Geriatr Oncol*. 2016;7:234–41. doi: 10.1016/j.jgo.2016.02.005

76 Pahor M, Kritchevsky SB, Waters DL, *et al.* Designing Drug Trials for Frailty: ICFSR Task Force 2018. *J Frailty Aging*. 2018;7:150–4. doi: 10.14283/jfa.2018.20

77 Rodrigues M, Oprea A, Johnson K, *et al.* Primary outcome reporting in clinical trials for older adults with depression. *BJPsych Open*. 2024;10. doi: 10.1192/bjo.2023.650

78 Bruner DW, Movsas B, Konski A, *et al.* Outcomes Research in Cancer Clinical Trial Cooperative Groups: The RTOG Model. *Qual Life Res*. 2004;13:1025–41. doi: 10.1023/B:QURE.0000031335.02254.3b

79 Young HM, Miyamoto S, Tang-Feldman Y, *et al.* Defining Usual Care in Clinical Trials. *Res Gerontol Nurs*. 2020;13:125–9. doi: 10.3928/19404921-20191127-01

80 Caraceni A, Cherny N, Fainsinger R, *et al.* Pain Measurement Tools and Methods in Clinical Research in Palliative Care: Recommendations of an Expert Working Group of the European Association of Palliative Care. *J Pain Symptom Manage*. 2002;23:239–55. doi: 10.1016/S0885-3924(01)00409-2

81 Witham MD, Stott DJ. Conducting and reporting trials for older people. *Age Ageing*. 2017;46:889–94. doi: 10.1093/ageing/afx153
